# Simulating Deforestation in Minas Gerais, Brazil, under Changing Government Policies and Socioeconomic Conditions

**DOI:** 10.1371/journal.pone.0137911

**Published:** 2015-09-15

**Authors:** Kayla Stan, Arturo Sanchez-Azofeifa, Mário Espírito-Santo, Carlos Portillo-Quintero

**Affiliations:** 1 Department of Earth and Atmospheric Sciences, 1–26 Earth Sciences Building, University of Alberta, Edmonton, Alberta, Canada; 2 Department of General Biology, CP 126 CEP 39401–089, State University of Montes Claros Montes Claros-MG, Brazil; University of Guelph, CANADA

## Abstract

Agricultural expansion is causing deforestation in Minas Gerais, Brazil, converting savanna and tropical dry forest to farmland, and in 2012, Brazil’s Forest Code was revised with the government reducing deforestation restrictions. Understanding the effects of policy change on rates and locations of natural ecosystem loss is imperative. In this paper, deforestation in Minas Gerais was simulated annually until 2020 using Dinamica Environment for Geoprocessing Objects (Dinamica EGO). This system is a state-of-the-art land use and cover change (LUCC) model which incorporates government policy, landscape maps, and other biophysical and anthropogenic datasets. Three studied scenarios: (i) business as usual, (ii) increased deforestation, and (iii) decreased deforestation showed more transition to agriculture from shrubland compared to forests, and consistent locations for most deforestation. The probability of conversion to agriculture is strongly tied to areas with the smallest patches of original biome remaining. Increases in agricultural revenue are projected to continue with a loss of 25% of the remaining Cerrado land in the next decade if profit is maximized. The addition of biodiversity value as a tax on land sale prices, estimated at over $750,000,000 USD using the cost of extracting and maintaining current species ex-situ, can save more than 1 million hectares of shrubland with minimal effects on the economy of the State of Minas Gerais. With environmental policy determining rates of deforestation and economics driving the location of land clearing, site-specific protection or market accounting of externalities is needed to balance economic development and conservation.

## Introduction

Deforestation in tropical regions is caused by economic growth and the expansion of industries such as agriculture and mineral extraction [1–3]. These drivers also commonly impact regional social and economic development by affecting natural capital, soil erosion, salinization, and carbon stock changes [4–6]. Where agriculture is the primary reason for ecosystem destruction, slash-and-burn practices are often employed to clear land for crops and pastures [1, 7, 8]. Fragmented, undeveloped areas of forest often dictate the landscape recovery time because higher exposure to anthropogenic activities contributes to edge degradation, stress of patch interiors, and structural changes [6, 9–11].

Landscape alteration models have focused on biophysical factors, including climatic variability, ecosystem stability, and degradation of the natural biomes [5, 12, 13]. Deforestation drivers and consequences have been projected into the future with these models relying heavily on accurate land cover maps [2, 14, 15]. Geoinformation, showing details about biophysical, socioeconomic, and population factors, are also important for predicting future locations of change. Field measurements of geographic information are highly accurate but often have gaps due to the time-consuming and inconsistent methods of data collection [16]. Remote sensing and GIS mapping procedures have significantly improved regional landscape mapping and enhanced models by incorporating socioeconomic variables [17–19].

Spatially explicit models of Brazilian biomes which combine land cover change, policy, and economic modeling are uncommon outside of the Amazon, with an information gap specifically in semi-arid environments such as those found in the north of the State of Minas Gerais. There has also been modeling of environmental policy, by Sparovek et al [17, 18], studying the overall effects of the Forest Code in Brazil. While public opinion is favorable towards environmental policies in Brazil, with a significant positive correlation between environmental policy and higher education [20], there remains heavy deforestation and land use change. This trend is due to a lack of incentives for farmers to protect natural areas on their land, especially with reduced penalties for non-compliance with current conservation legislation [21, 22]. However, even with little research into future projections, there is still the ability to model which economic drivers must be enacted to initiate conservation.

The Brazilian Forest Code is a set of federal laws which regulate the amount of original biome that must be kept intact on new farms [23], and minimum buffer zones to protect riparian areas and mountain regions from deforestation [23]. The Code was recently repealed for a new law which has the potential to increase the overall degradation by reducing the restrictions on locations of deforestation and decreasing penalties for deforestation prior to 2008 [21, 24]. The legislation allows landowners to pay to conserve an equivalent value of land elsewhere in the biome instead of on their own property [21, 24]. The amendments made to the policy may contribute to decreased deforestation in the Amazon; however, other researchers suggest the reverse is likely to be true in the Cerrado and Caatinga [24].

In Minas Gerais, Brazil, past deforestation research focused on three primary objectives: 1) State-wide vegetation mapping (a forest inventory) to determine the distribution of natural vegetation and planted forests, as well as to estimate vegetation structure and monitor deforestation [25]; 2) localized vegetation mapping [26]; and 3) biome-specific modeling [5, 13, 16, 27]. Research on land use/cover change mapping has taken place in different regions of the state, usually at specific parts of hydrographic basins or relief formations [26, 28–31]. Biome-specific modeling focused on remnant vegetation and effects of land cover changes on the earth systems [5, 13, 16, 27]. Ribeiro et al [16] and Hirota and Ponzoni [32] were interested in fragmentation and conservation prospects for the Atlantic Forest, while Rocha et al [33] and Sano et al [34] mapped land use and land cover for the Cerrado biome in different periods. Deforestation in both Cerrado and Caatinga biomes were recently estimated by Beuchle et al [35]. Batlle-Bayer et al [5], Brannstrom et al [27], and Carvalho et al [13] also studied the ecosystem functions and changes in the Cerrado biome. These studies provided a holistic view of the biome using previous literature, remote sensing, government maps, and computer modeling.

Studies in Minas Gerais have not focused on how government legislation and changes in economic conditions impact the natural environment using a quantitative, spatially explicit approach. Lack of attention to the effects on land cover change produced by socioeconomic variables and legislation has been due to a deficiency in spatially explicit data and problems linking qualitative data to biophysical models [12]. The Dinamica EGO platform attempts to reconcile the data differences by allowing input of biophysical and economic variables while providing an avenue for implementing policy changes [36]. This integrated approach can be used to promote sustainable growth by balancing ecosystem protection and economic efficiency.

The feasibility of starting a project which may affect land cover change can be determined by cost-benefit analysis, which compares total costs with potential financial gain [37, 38]. Cost-benefit analysis optimizes profit and assumes the presence of capitalism, no externalities, and that a competitive market is present in the area of study [38]. Ecosystem services are externalities that are unaccounted for by this traditional analysis and, therefore, must be internalized in the markets by attaching value to the services.

Projecting economic decisions is typically completed separately from spatial models utilizing mathematical programming [4, 39], and agricultural systems are best computed using linear optimization models. These models are similar to cost-benefit analysis by attempting to optimize profit, in turn offering insight into the future financial repercussions of changing the landscapes. When integrated with projection-based spatial models, they can be used to balance both profit and conservation. Equilibrium between economic development and preservation of natural capital is needed to optimize support from government, public interest groups, and industry [6, 15].

This study aims to combine biophysical, legislative and socioeconomic data to model the changes in the Minas Gerais landscape and project them into the future. An approach focusing on data integration can be utilized to explore the policy and economic options which may be implemented for sustainable growth. This paper also explores the effect of adding the natural value of the land into an economic model to predict how full cost accounting can be used to measure the impact on rates of deforestation and the allocation of agricultural land in semi-arid regions of Brazil. Models were run annually until 2020 (spatial) and 2023 (economic) to determine the effect of policy and biodiversity value on the landscape using two different methodologies.

## Materials and Methods

### Study Region

The study area is the Brazilian State of Minas Gerais, an interior region with an area of over 586 000 km^2^ and more than 20 million people. According to the Ministério do Meio Ambiente (MMA), Minas Gerais is comprised of three major biomes, namely the Mata Atlântica, Cerrado and Caatinga [40, 41]. Tropical Dry Forests, a particularly sensitive vegetation type, are split between the three biomes and are prevalent in the northern part of the state [42]. The Cerrado has a total area of 2.04 million square kilometres; however, less than 50% of the original ecosystems are still intact [40, 43]. Mata Atlântica is located in the southeast of the state and, at most, 25% of this biome remains in extremely fragmented zones due to extensive urban development [40, 43]. Between 30% and 50% of the Caatinga, found in northern Minas Gerais, has been altered by humans [43]. The Mata Atlântica has just over 10% of its remaining fragments protected in natural reserves or protected areas, whileonly 2.2% of the Cerrado land and 1% of the Caatinga are in designated protected regions, and the remainder is allowed to be changed for anthropogenic uses [16, 43, 44]. Those figures do not include the 20% of natural land that is mandated to be protected on all farming areas located in Cerrado and Caatinga [21].

**Fig 1:**
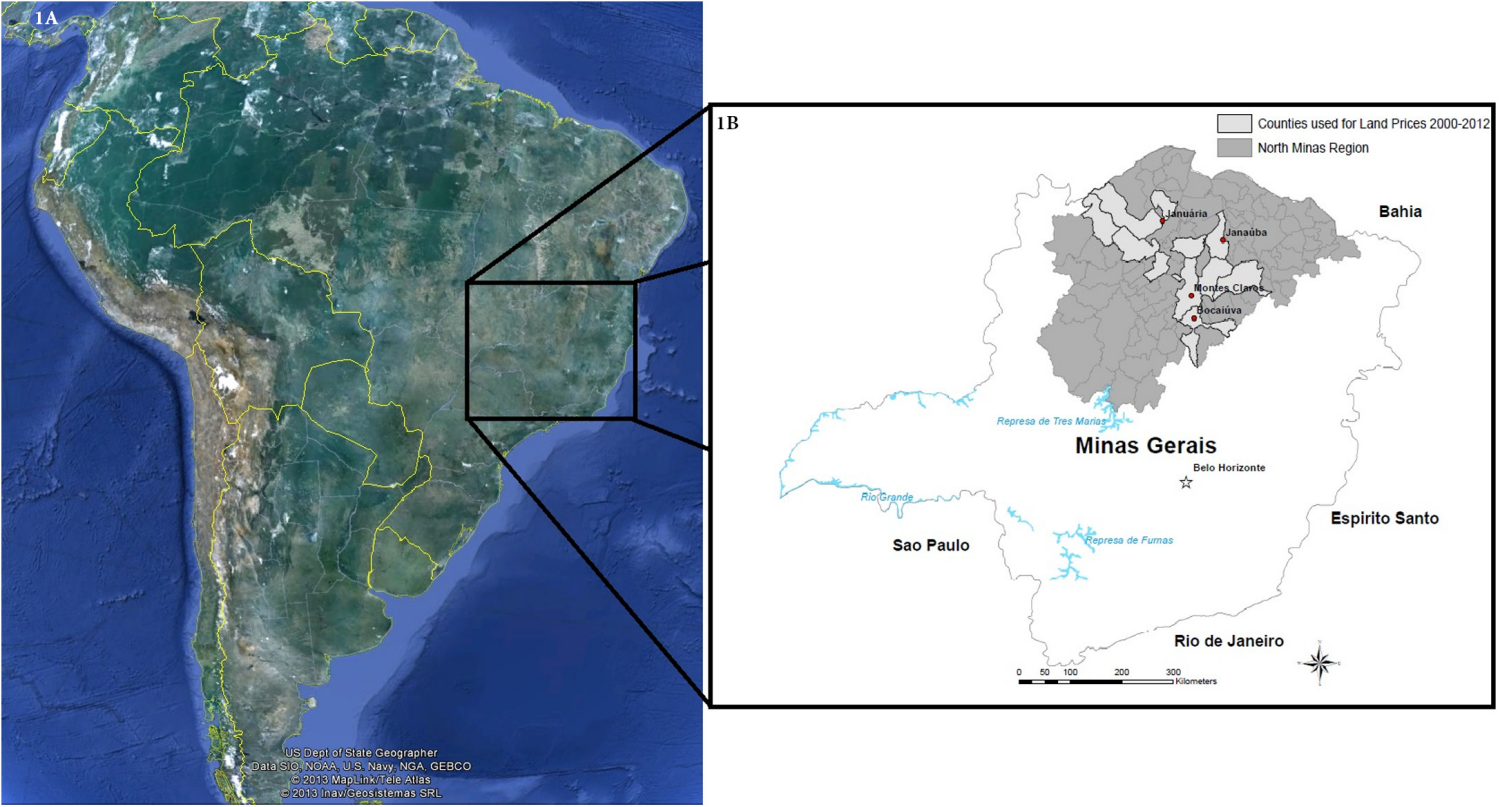
Study site is the State of Minas Gerais, an interior state in Brazil. County land prices are taken from the northern region of this state. Left: Reprinted from Google Maps under a CC BY license with permission from Google,2013; Right Image compiled using the Dinamica EGO software using political borders available from the IBGE, 2015.

The entire State of Minas Gerais was utilized for both the spatial and economic models, with the landscape divided by land cover type in the spatial model and by biome in the economic model. Land prices from 2000–2012 were derived from the north of the state (Fig 1) and then extrapolated for an average sale price for the entire state.

### Spatial Model Data

For the model, two land cover maps were taken from 1994 and 2000, using the Global Land Cover Facility (University of Maryland) and European Commission Joint Research Centre datasets, respectively. The University of Maryland 1994 map originally had 14 vegetation categories derived from Advanced Very High Resolution Radiometer (AVHRR) satellite images [45]. The European Commission Joint Research Centre classified images had information derived from SPOT 4, Along Track Scanning Radiometer (ASTR)-2, JERS-1 radar and DMSP (Defense Meteorological Satellite Program) that was divided into 22 categories, with the higher differentiation possible initially because of the data integration [46]. The classification system of both maps is based on phenology not biome and the resulting designations are based on vegetation type. The methodologies for categorizing land cover were different for each organization, leading to large data errors if these different datasets were compared as they were produced. As a result, for this study, the land cover maps were reduced to forest, shrubland, and agriculture, with forest defined as any area with a majority (>50%) of trees, shrubland as all other natural landscapes including grasslands and areas with few to no trees, and agriculture as any anthropogenically altered or managed landscapes. Both maps had a 1 km spatial resolution and were consolidated to include only broad categories of forest, shrubland, and agriculture to account for the different classification methodologies and inherent data error.

All additional data used for tuning the model were acquired from the Instituto Brasileiro de Geografia e Estatística (IBGE). These included biophysical variables, climate, soil fertility and type, vegetation, and relief, derived from 2012 thematic maps (http://bitly.com/1C7UdNM), population from the 2010 census (http://bit.ly/1H0pTrn), and road maps from the Brazilian Ministry of Transport.

### Modeling Scenarios and Parameters

The Dinamica EGO platform is utilized to complete a comprehensive land cover change model by determining the probability of each individual cell changing from one land cover to another [47]. Landscape maps are input to calculate a transition matrix that outlines annual rates of change between each cover type. Variables, including any relevant spatially explicit datasets, are used to identify where change is most likely to occur. The probability is calculated by giving each variable a relative importance (weight of evidence) based on a Bayesian algorithm that identifies probabilities of transition compared to the categories on the static variable maps [47]. If expert opinion disagrees with the algorithm output, the weights can be manipulated to better express current trends.

**Fig 2:**
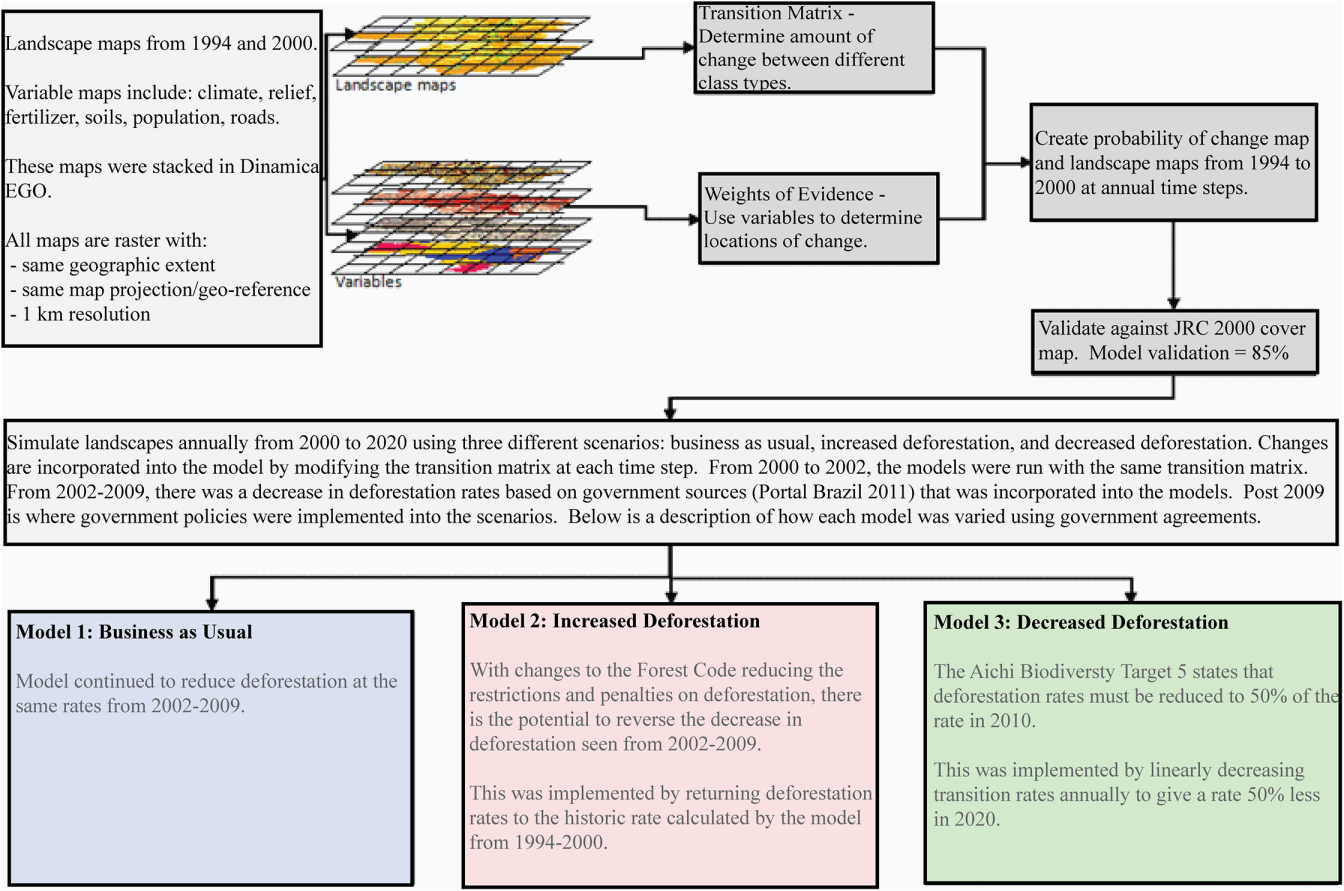
Model description and simulation data inputs, parameters, and workflow.

A probability of change map is created for each land cover, and the probability of each pixel being altered is determined through the weights of evidence. Two functions, Patcher and Expander, are used to train how continuous and connected the new islands of land cover type will be. The pixels with the highest probability of alteration, which comply with the conditions set by Patcher and Expander, will change. In this study, the model projected the landscape for the state from 1994 to 2000 and compared the results to the JRC image to validate the model performance. Following model validation, the entire state landscape was projected into the future from 2000 to 2020 using legislation to train rates of change and variable importance. Three scenarios were simulated: i) business as usual (BAU), ii) increased deforestation using the changes to the Forest Code, and iii) reduced deforestation using the Convention on Biological Diversity Aichi Biodiversity Targets (Fig 2).

The BAU scenario continued historical trends and accounted for the reduction in deforestation rates reported by the MMA between 2002 and 2009 (http://bit.ly/1JPGBG1). The second scenario was based on revisions to the Forest Code of 1965, where the potential increase in deforestation, due to reductions in land alteration restrictions and penalties for non-compliance in the new Code, was modeled. An increase in the rate of change was input into the model, and the weights of evidence were modified based on the reductions to the restrictions in certain zones. Any change in the amount of land altered was a function of the rate change, and differences in the spatial distribution of land cover were due to the modified weights of evidence. Brazil has also signed onto the Aichi Biodiversity Targets from the Convention on Biological Diversity, which states that signatory countries must reduce deforestation by 50% by 2020 (Target 5) [48, 49]. The third model predicted the effects of this environmentally favorable initiative by reducing deforestation trends annually between 2011 and 2020 (Fig 2).

The resulting maps were regionalized into 25 km x 25 km sectors to compare the probability of change to the percentage of remaining natural ecosystem, and to compare biome loss to agricultural gain in each area. The 25 km resolution allowed for an area just over the mean size of municipalities (counties) in Minas Gerais (614 km^2^), allowing whole political units to be compared overall. The standardization of size was preferable to comparing the political designations, as the ecosystems are not constrained by political boundaries. Further, fragmentation statistics, including the minimum, mean, and maximum patch sizes, were also calculated for each cover type.

### Economic Model

The simplest way to mathematically model the economic trade-offs in a farming system is through linear programming, which maximizes a profit function subject to a set of linear constraints [47]. Constraints include crop yields and maximum areal extents, labour requirements, and the capital available for investment. Some of the technologies and resources may not have linear relationships; however, assuming a well-behaved, non-linear function is present, the constraint can be approximated by a set of linear equations [47]. Modeling biodiversity is not frequently completed using this simplistic system. Instead, biodiversity is typically considered through the presence or absence of individual species in a region or by the inherent uncertainty present in ecosystem management, binary and risk programming, respectively [50, 51]. These two economic models require extensive data input and are difficult to interpret. As a result, based on data availability and the objectives of this study, which include deriving the area converted from the natural landscape (with and without the inclusion of a biodiversity tax) as an explicit value, a linear programming model was used instead of binary or risk programming. Linear models, although simple, result in explicit expressions of monetary values and areal extents of land bought, making them easily communicable to producers and policy makers.

A linear optimization model was used to determine the optimum area (in ha) of the five most common crops in Minas Gerais and to project the revenue generated based on historical price and yield records. Due to a lack of data and the spatially constrained nature of the information, the costs associated with technology (i.e. fertilizer, machinery, etc.) were not incorporated into the model. Profit was therefore optimized by maximizing the difference between the costs of growing each crop (labour and acquiring land) and the revenue generated from each crop (price, yield, and areal extent). The outputs of this model included the resulting profit generated, the optimal total area for each crop, the amount of labour needed, and the land bought. A linear optimization model iteratively goes through different combinations of the above output variables, and the output results are found when the profit is the highest that can possibly be derived given the cost constraints.

The maximization of profit over time derived from the crops was represented by:

$$Max\;\pi =\sum_{t=0}^{14}df\sum_{i=1}^{5}P_{i,t}X_{i,t}-J\;*\;L_{t}-K_{t}\;*\;R_{t}$$

(1)where Π = profit derived from the regional agricultural scheme in Minas Gerais ($ USD); *df =* the discount factor which accounts for currency inflation; t = number of time steps in the model (year number), t = 2010–2023; i = crop type: 1 (beans), 2 (cane sugar), 3 (coffee), 4 (corn), and 5 (soybeans); P_i,t_ = price associated with each type of crop over every time step–this is a linearly increasing function derived from historical values ($ USD/ha); X_i,t_ = the area of each crop “i” in each time step “t” (ha); J = the minimum wage associated with farming ($ USD/day); L_t_ = the total amount of labour required to be hired to work the agricultural land (days); K_t_ = the price of buying new farmland–this is a linear function derived from land sale prices from 2000–2012 in northern Minas Gerais ($ USD/ha); R_t_ = total area bought for farmland conversion in a time step.

The maximization problem is constrained with the following associated assumptions:

$$\begin{array}{l} df=\frac{1}{\left(1+\text{inflation rate}\right)}\\ X_{i,t}=X_{i,t-1}+R_{i,t-1}\\ X_{i,t}\leq X_{i,t-1}\;*\;G_{i}\\ \sum_{i=1}^{5}R_{i,t}=R_{t}\\ \sum R_{t}\leq \text{Total Natural Land in Minas Gerais}\\ \sum_{i=1}^{5}L_{i,t}=L_{t} \end{array}$$ (1)

Where X_i,t-1_ = the total area of each crop “i” in the previous time step, t-1; R_i,t-1_ = the area bought in the previous time step “t-1” for each crop type “i”; G_i_ = the historical rate of expansion for each crop type “i”; and L_i,t_ = the amount of land required to grow and maintain each crop “i” during time step “t.”

The model was slightly modified to project the importance of natural capital by incorporating the cost of biodiversity into the price of land purchased in each biome. In this model, the same constraints and assumptions of maximizing the difference between cost and revenue still apply. The difference between this and the previous model is that the K*R_t_ term has been expanded to include the specific biomes that could be converted–Cerrado/*Cerr*_*t*_ (ha), Caatinga/*Caat*_*t*_ (ha), Mata Atlântica/*MA*_*t*_ (ha), and the cost of buying each area if the biodiversity value from each ecosystem type was included as a tax, i.e. K_t_ + biodiversity cost Cerr/Caat/MA = K_Cerr_/K_Caat_/K_MA_:

$$Max\;\pi =\sum_{t=0}^{14}df\sum_{i=1}^{5}P_{i,t}X_{i,t}-J\;*\;L_{t}-K_{Caat}\;*\;Caat_{t}-K_{Cerr}\;*\;Cerr_{t}-K_{MA}\;*\;MA_{t}$$ (2)

Crop areas, as well as yields and revenue by crop type were gathered for Minas Gerais from 2003–2010 to train the linear cost constraints and project growth rates, and were collected from the IBGE database (http://www.sidra.ibge.gov.br/). The sale price of farmland ($ USD/ha) was derived from data collected from the Banco do Nordeste do Brasil. Labour requirements (days by crop type) to grow and harvest crops were obtained through literature [52–55]. Beans and corn labour data were gathered from the Latin American Studies Association [52], coffee from Vosti et al [53], cane sugar from Hermele [54], and rice from Thompson and Blank [55]. The amount of free labour in the state and the minimum wage were acquired from the 2006 Agricultural Census (IBGE 2006). Both economic models include the entire state and do not regionalize results.

### Species Value

The value of individual species was calculated by Resende et al [56], specifically for the Serra do Cipó mountain range in Minas Gerais. These values were calculated as the cost of maintaining the genetic diversity of all current species ex-situ. The price of managing species, including the costs of finding, transporting, and caring for the plants was collected from the Zoo-Botanical Garden. The resulting value of species was between $5,148 and $7,819 for common species, and between $51,475 and $78,186 for endemic or endangered species. To extrapolate the values from Resende et al [56], the total number of known plant species in Minas Gerais was collected through the Botanical Garden of Rio de Janeiro, and their status as endemic, endangered, or common was retrieved through a combination of the Botanical Garden of Rio de Janeiro database and Biodiversitas. This methodology is useful because it provides an objective view of the biodiversity value that is not subject to changing societal values.

## Results

### State-wide Spatial Simulation Scenarios

**Fig 3:**
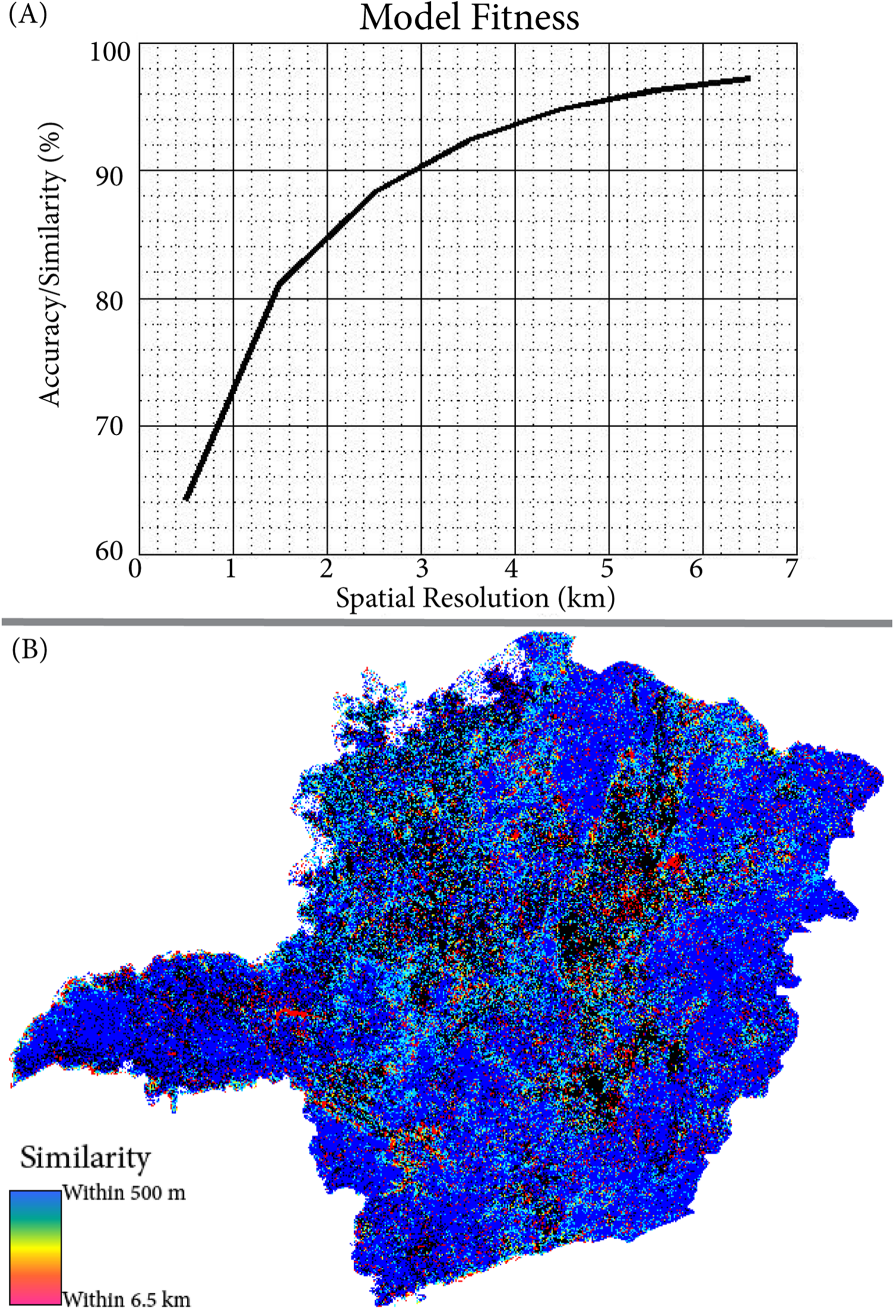
(A) The model validation curve comparing the similarity of the model result for 2000 to the JRC classified map. (B) The spatial distribution of which patches validate well. Overall, the larger patches validate better than the smaller ones.

**Fig 4:**
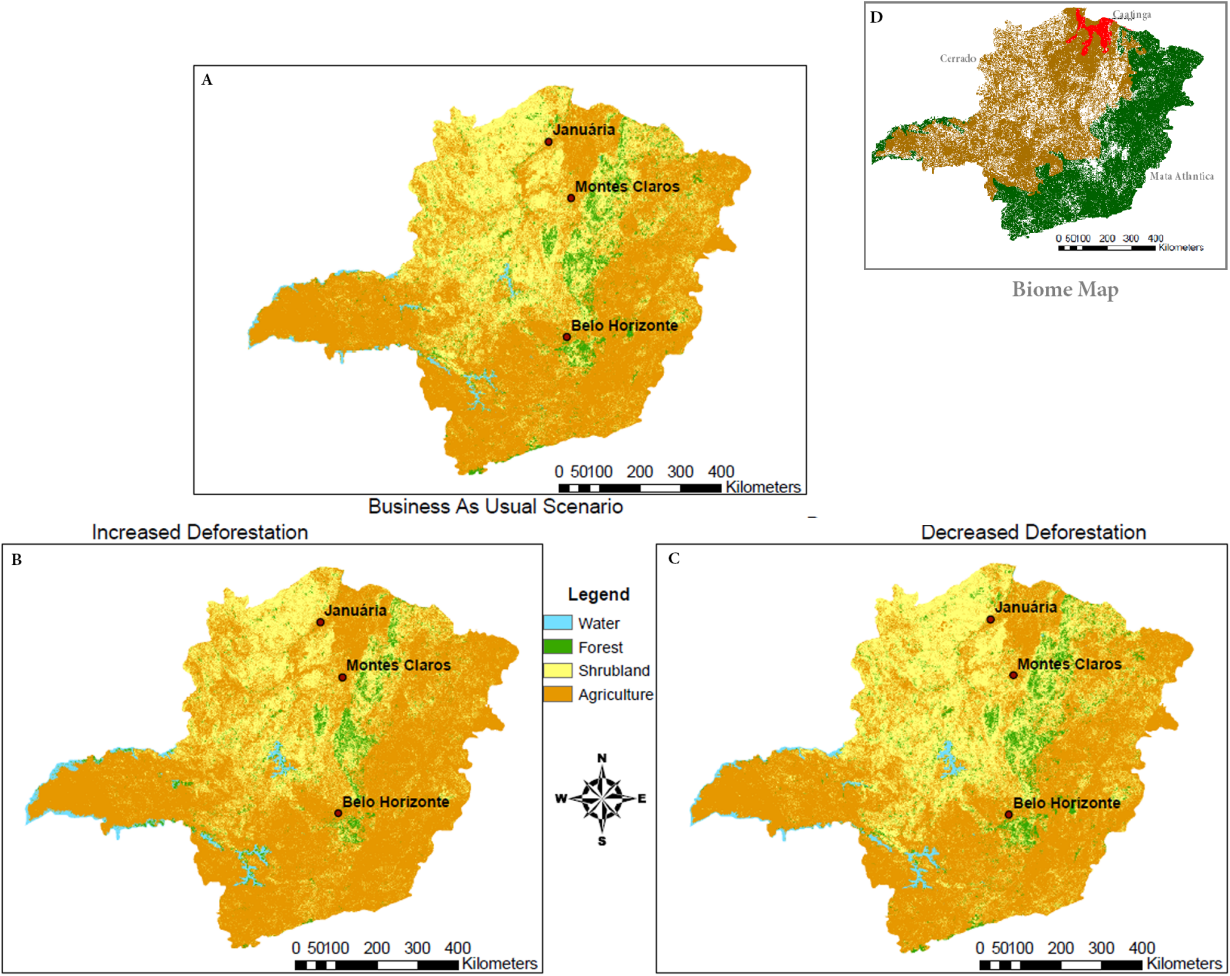
Results from Dinamica EGO model simulation showing the landscape composition for the year 2015. The scenarios run were business as usual (A), increased deforestation from the Forest Code revisions (B), and decreased deforestation rates from meeting the Aichi Biodiversity Target (C). A biome map (D) is included for a point of reference of where the main changes occur by biome (Map freely available from the IBGE geographic datasets). Image reprinted under a CC License with permission from Kayla Stan, 2015.

The spatial model, which predicts land cover including forest, shrubland, or agriculture, was validated to be over 85% accurate within 1.5 pixels, and this value increased to over 90% within 3.5 pixels (Fig 3A). Larger fragments of a single cover type validated better than highly heterogeneous areas (Fig 3B). All three scenarios, business as usual, increased deforestation, and decreased deforestation, are most strongly impacted by deforestation in the central and northwest regions (Fig 4). Shrubland is consistently and preferentially deforested in these models, and is influenced by policy changes more strongly than the forest. The Forest Code revisions result in less natural land remaining and higher fragmentation for both natural cover types. The reduction in the amount of natural land is a function of the increased rates of deforestation implemented by the model while the spatial distribution or fragmentation statistics is a function of changes in the weights of evidence, based on the modification of restrictions of land conversion in the Forest Code. Meeting the Aichi Biodiversity Target, decreased deforestation scenario, leaves the most original ecosystems intact and preserves larger patches.

There are a large number of the small forest patches cut down entirely in the increased deforestation prediction scenario. This scenario also has a substantial reduction in the size of the largest patches over time, which, when combined with the reduced number of small patches, leads to an increased mean patch size with a smaller standard deviation (Table 1). The decreased deforestation scenario has the largest maximum patch size of all three scenarios, while the smallest reduction in maximum patch size between 2015 and 2020 occurs in the BAU scenario. Shrubland cover shows a different trend with both the smallest mean and maximum patch sizes found in the increased deforestation scenario, and the largest mean and maximum patch sizes from the Aichi Biodiversity Target scenario.

**Table 1:** Landscape fragmentation parameters in 2015 and 2020 for each scenario and land cover type. Parameters include both the mean and maximum patch sizes.

	Forest Patch Sizes (km²)				Shrubland Patch Sizes (km²)			
	Mean 2015	Mean 2020	Max 2015	Max 2020	Mean 2015	Mean 2020	Max 2015	Max 2020
Business as Usual	4.3	4.4	2,380	2,070	12.5	12.2	140,000	140,000
Increased Deforestation	5.6	5.7	1,670	820	3.6	3.4	1,370	1,360
Decreased Deforestation	4.6	4.8	3,950	2,510	14.1	16.3	183,000	159,000

**Fig 5:**
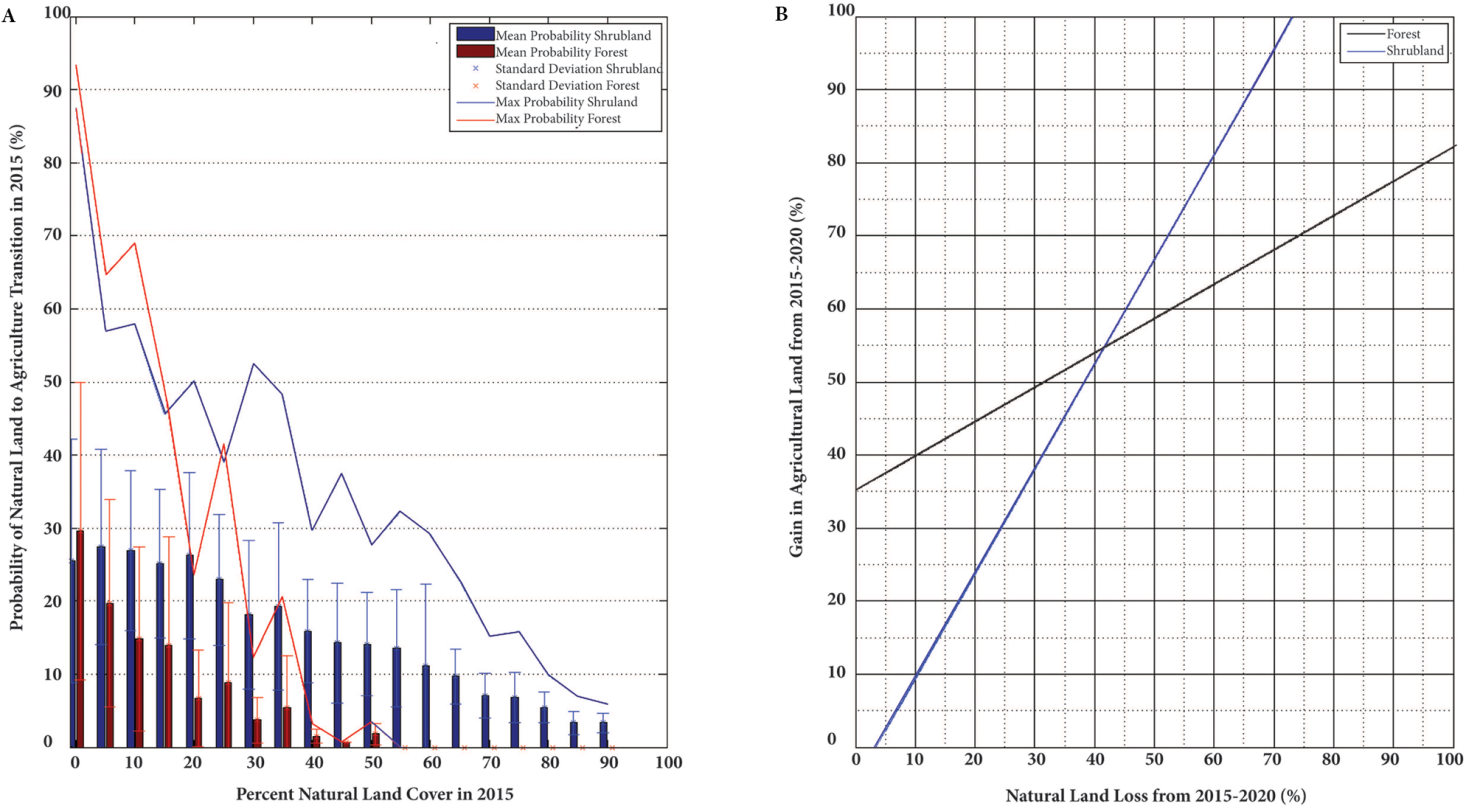
The probability of transition compared to the amount of original biome remaining (A), and the comparison of natural biome loss to agricultural gain (B).

This discrepancy may be explained by the comparison of the probability of transition and the amount of natural biome remaining in a region. The probability of change is highest when the remaining forest cover is 0–5% and exponentially decreases with increased forest cover in an area (Fig 5A). The shrubland change probability decreases linearly as its cover extent increases. It also has a higher mean probability of change with more than 5% shrubland cover in a region when compared to the equivalent extent of forest. Both forest cover and shrubland loss have a positive association with agricultural gain (Fig 5B). As these natural ecosystems decrease in size, there is a corresponding gain in cropland, with shrubland having a sharper increase when compared with forest loss (Fig 5B).

### State-wide Economic Simulation Scenarios

**Fig 6:**
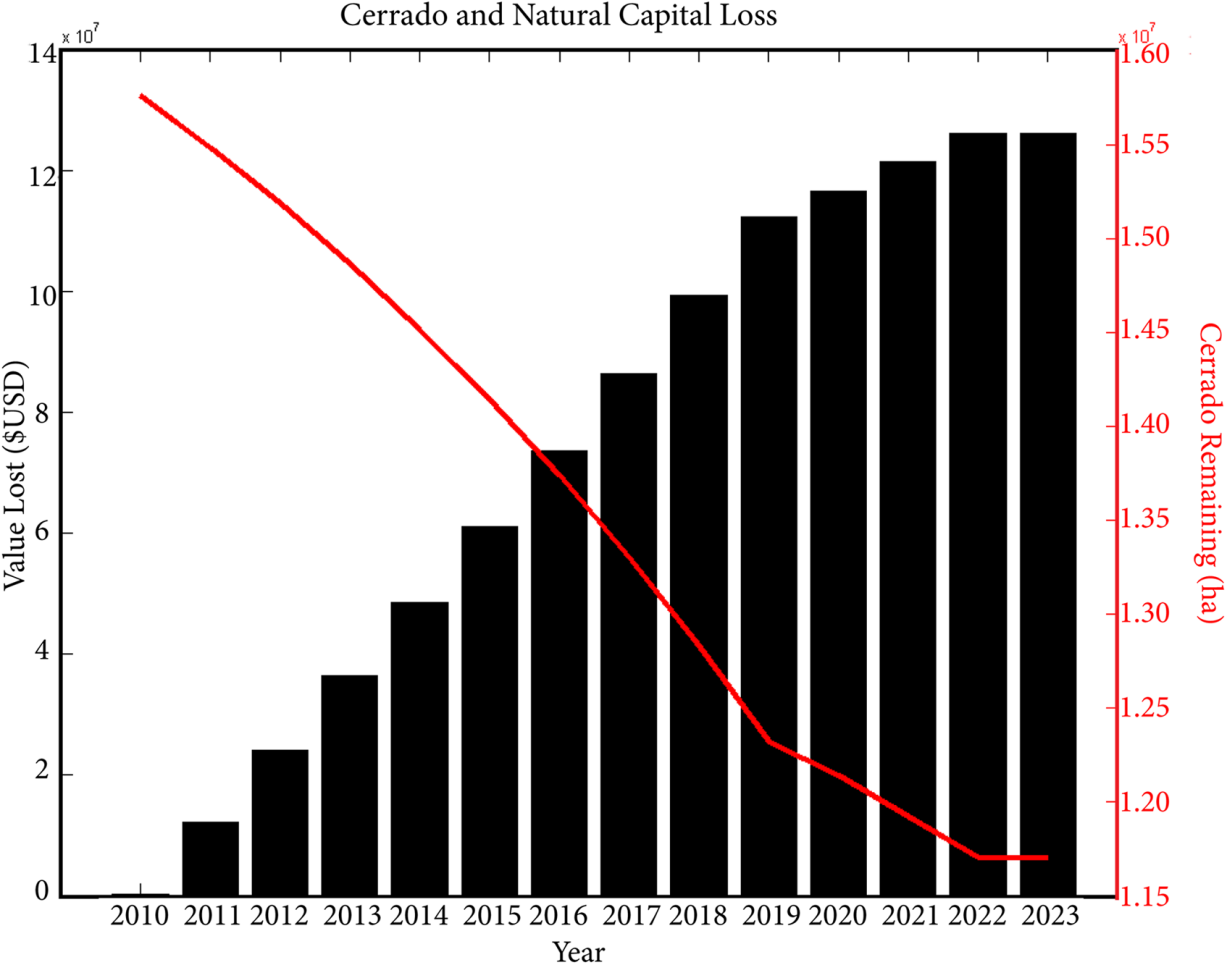
The remaining Cerrado land and the cumulative value of the Cerrado deforested between 2010 and 2023.

**Fig 7:**
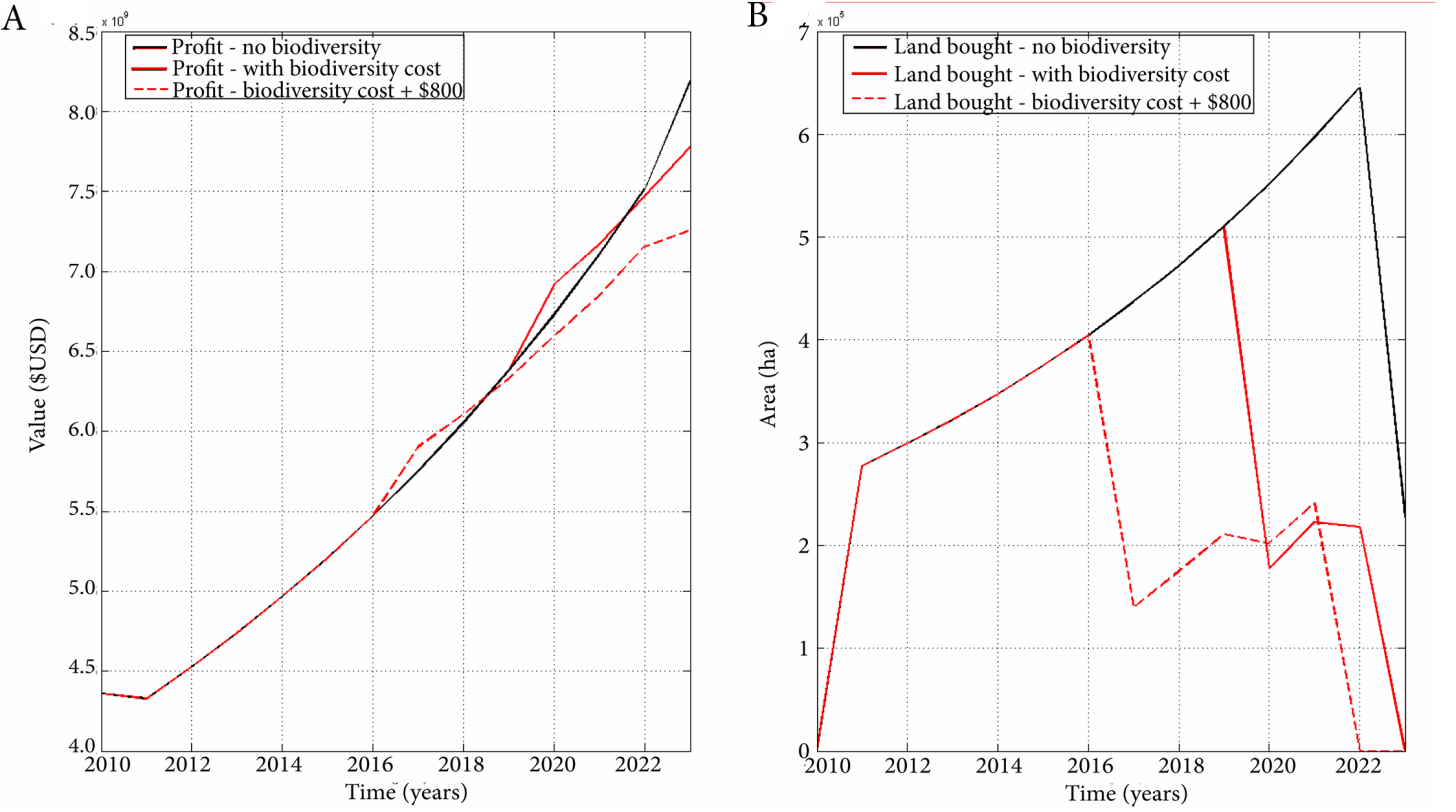
(A) The annual profit from the no-biodiversity model and the optimization scenario with biodiversity as part of the variable costs; (B) The amount of land bought per year in the original and biodiversity models.

Minas Gerais’ landscape in the economic models is divided by biome, not land cover as in the spatial model, and our results illustrate that, if profit is maximized with no other assumptions of risk or geographic constraints, the remaining Cerrado area in 2010 decreases by 4 million hectares, leaving only 11.7 million hectares intact by 2023 (Fig 6). Profits from agriculture generated in each year increase from $4.4 billion USD in 2010 to over $8.2 billion USD in 2023 in the first resource allocation model (Fig 7A–black curve). This model, which does not account for biodiversity costs, predicted that the amount of land bought annually will increase up until 2022 and decrease in 2023 (Fig 7B–black curve). This decrease in the final year may be due to the discount factor (inflation) having sufficiently outpaced the revenue costs, making it less profitable to buy land.

The biodiversity values calculated from Resende et al [56], led to an overall natural land value in Minas Gerais of over $750 million USD (Table 2). The biodiversity values of each biome were divided by their areal extent and these values per hectare were used as the K values in the optimization model which included the biodiversity constraints.

**Table 2:** The value of land as determined by using an average value per species.

Biome	Value	Value per ha
Cerrado	364,074,657	1,743.68
Mata Atlântica	303,438,607	1,791.67
Caatinga	82,972,053	1,995.96
**Total ($USD)**	**750,485,317**	

The biodiversity scenario resulted in a cumulative net profit of $53.04 billion USD from agriculture in the 13 year period, just $2.5 billion dollars less than the first model, with only a small reduction in annual profit after 2020 when comparing the two models (Fig 7A–solid red curve). By including biodiversity in the model, through the price of land sales, there was no immediate loss in profit and only a 5% revenue decrease by 2023, a total reduction of 0.3% of the GDP growth (Fig 7A–solid red curve). In that 13 year period, over $125 million USD would be collected specifically from the biodiversity value that was added to the land sale price as a tax. This ecosystem tax would conserve over 1 million hectares of Cerrado land (Fig 7B–solid red curve), allowing for continued export of natural capital as well as other benefits, including reduced salinization and increased stability of soils. The conservation effects observed through the addition of a biodiversity tax could be further improved with increasing the taxation amount. With a larger tax, more land protection could occur and more tax revenue would be generated, but profits derived from farming would take a further and more substantial cut (Fig 7A/7B–dashed red line).

Of the three biomes in Minas Gerais, the model indicated that it was most cost effective to alter the Cerrado land. Both the Caatinga and Mata Atlântica would remain untouched if the optimal allocation of land was considered with biodiversity value. It would cost an additional $115 to $252 per hectare to buy Caatinga over Cerrado land, and an additional $22 to $136 to buy a hectare of Mata Atlântica.

## Discussion

The policies included in the model scenarios are not location-specific, instead focusing on regional rates of deforestation, resulting in much alteration of the northwest in all scenarios run. The biophysical variables, therefore, dominate the calculations of probability of deforestation in each location in the model. These results agree with the optimization model and typical economic theories which assert that the benefits associated with an activity must outweigh the production costs to make the project profitable [37]. There are less overhead costs associated with clearing shrubs compared to cutting forests for agriculture, which is consistent with Cerrado being preferentially deforested when allocating resources. The high probability of conversion to agriculture is correlated with the lowest percentages of remaining natural ecosystems (Fig 5). This would imply that new land alteration will be in close proximity to other farming operations, consolidating the revenue base and decreasing transportation costs between plots of land. Different government policies more strongly affect shrubland compared to forests in the Forest Code revisions and Aichi Biodiversity Target models.

The exponential decrease in forest transition with increased land cover will result in the disappearance of the smallest forest patches because they are being preferentially cut. This trend of cutting the smallest forest patches results in an increase in the mean forest ecosystem, a tendency that is exhibited in all three models between 2015 and 2020. Shrubland clearance is more uniform because this cover type has a lower gradient of mean probability of transition, resulting in a decrease in mean patch size with the BAU model and Forest Code revisions compared to the Aichi Biodiversity Target scenario. The decreased deforestation scenario shows an increase in the average patch size, possibly indicating secondary regrowth on abandoned lands.

The health and functionality of ecosystems are severely impaired with a reduction in patch size, abundance, and connectivity. Genetic diversity and re-colonization efficiency is improved and degradation is reduced in larger and more continuously connected patches [57]. In the case of Minas Gerais, with few patches remaining and high rates of deforestation projected in the near future, the functionality of the original ecosystems is expected to be reduced. Connectivity is projected to decrease in the state, limiting species dispersal and migration, thereby damaging these ecosystems irreparably [57].

In Minas Gerais, there is a corresponding gain in agriculture land when natural area is lost, with a higher conversion rate when considering large losses (>40%) in shrubland (Fig 5). Less preparation for clearing is needed in Cerrado which, in addition to local climatic and soil variations, accounts for high correlation, broad scale locations of clearing, and overall crop sustainability [58].

Other studies reached similar conclusions, finding that Cerrado deforestation rates are three times those of the Amazon basin, despite the long dry season and required soil modification because of low pH and excess aluminum [44, 58, 59]. Deforestation trends are related to both biophysical and economic forces, according to the studies, with location-determining factors including site conditions and proximity to anthropogenic alterations [60].

Minas Gerais has exhibited exponential increases in land sale prices in the past decade (Banco do Nordeste do Brasil), and the revenue from crops is projected to expand annually (IGBE), indicating that there is, and will continue to be, an increased ability and willingness of entrepreneurs to invest money in the farming industry. With increased investment, the threshold that limits growth rises and the optimal percentage of land to deforest can increase. This can be exacerbated by reduced costs and improved yields from technological advances, especially in soil modification and machinery. In the future, this may allow the trajectory of deforestation and fragmentation to increase beyond the model results.

Cost-benefit analysis, a foundation for the economic models in this study where the value of crops was compared to the cost of deforestation, is based on the first fundamental theory of welfare economics. This theory assumes there is the presence of capitalism, no externalities, and a competitive market in the area of study [38]. If these conditions are met, then there can be a natural optimization of financial surplus or profit [38]. There are flaws in applying these suppositions to agricultural scenarios, including market failure, imperfect competition and, more importantly, unaccounted for externalities [61, 62].

Externalities affect humid regions by reducing carbon stock held in the plant and soil communities, while soil erosion and salinization are more common in semi-arid ecosystems [6]. In Minas Gerais, soil erosion is prominent, washing fertilizers into nearby streams and decreasing water quality [44]. Slash-and-burn clearing releases large quantities of carbon into the atmosphere and often damages unintended areas [44]. Other externalities in this region include alterations to nutrient and water cycling [3], biodiversity loss, and expansion of invasive species [44]. As part of the Coase theorem, the producer and affected party must negotiate to come up with a financial arrangement that compensates for the impacted public goods or externalities [38].

In an attempt to account for these externalities, the value of biodiversity was characterized, estimated at $750,000,000 USD, and incorporated into one of the economic models. This amount, however, only encompasses one part of the natural capital value, while other externalities remain largely unquantified. If those rates were also determined, the value of the natural land would increase. The total Cerrado biodiversity price is the highest of all the biomes because of the large number of endangered and endemic species in the area. This value of Cerrado land is important because, according to the economic model, this biome will be preferentially cleared in the near future, given its large remaining extent compared to the other biomes, and only 2.2% is currently protected by law in Brazil [44]. At least 20% of the endemic and endangered species in this biome are not found on protected territory, and public and conservation attention to the Cerrado is much lower than the Brazilian moist forests, such as the Amazon and the Atlantic Rain Forest [63].

Globalization of economies stimulates land use conversion and Brazil’s emerging market makes it a strong contender to have accelerated changes on the landscape [3]. Alteration is hastened by high external demand based on increased connectedness and cash flow between geographically removed consumers and producers [3]. Most economic activities directly or indirectly use the land, but forestry and agriculture are some of the most involved industries [64]. Monitoring how the economy impacts the land and providing incentives that make conservation more affordable and profitable for farmers are crucial in growing nations such as Brazil [65]. Sustainable growth in the future relies on creating policies which balance public and social interests, conserve valuable sections of land, and increase stakeholder participation in policy development [65, 66].

Land use and cover change modeling has been useful to determine what future landscapes might look like and the economic impact of biodiversity; however, the problem remains that obtaining fine scale, reliable predictions is difficult [12]. Regions are dynamic, and the factors that determine the composition of future landscapes are rarely best represented solely by a static map [66]. Remote sensing data from satellites such as AVHRR, SPOT, or MODIS are commonly used, as in this study; however, coarse resolution data derived from these satellites can only track drastic changes while fine scale changes remain unknown [6]. These satellites have the largest datasets and are run for the longest time frames, with higher resolution satellites only increasing in popularity in the past 5–10 years.

Integration of socioeconomic data and increasing the ability to manipulate transition matrices over the temporal scale have improved with the Dinamica EGO platform compared to previous systems [6, 12, 47]. It is still extremely difficult to account for all of the variables that affect the transformation of landscapes over time; however, this system has proved to be useful in not only modeling Amazonia but also the Cerrado land and other semi-arid environments, making it a valuable transnational modeling platform.
